# BEAT-BK: An Adaptive, Randomized Controlled Trial to Treat Polyomavirus Infections (BKPyV) in Kidney and Kidney-pancreas Transplantation Recipients (BEAT-BK) Study Protocol

**DOI:** 10.1097/TXD.0000000000001924

**Published:** 2026-03-04

**Authors:** Ryan Gately, Michael Dymock, Dharshana Sabanayagam, Scott B. Campbell, Jonathan C. Craig, Vikas Dharnidharka, Anna Francis, Jonathan M. Gleadle, Martin Hajek, Sana Hamilton, Martin Howell, Nicole Isbel, Allison Jaure, David W. Johnson, John Kanellis, Karen Keung, Siah Kim, Jen Kok, Dirk R. Kuypers, Nicholas Larkins, Wai H. Lim, Rahul Mainra, Julie A. Marsh, Gabor Mihala, Brian J. Nankivell, Samantha Ng, Helen Pilmore, Nicole Scholes-Roberton, Thomas Snelling, Armando Teixeira-Pinto, Andrea Valks, Kenneth Yong, Carmel Hawley, Germaine Wong

**Affiliations:** 1 Department of Kidney and Transplant Services, Princess Alexandra Hospital, Brisbane, QLD, Australia.; 2 Wesfarmers Centre of Vaccines and Infectious Diseases, The Kids Research Institute Australia, Nedlands, WA, Australia.; 3 School of Population and Global Health, The University of Western Australia, Nedlands, WA, Australia.; 4 Sydney School of Public Health, University of Sydney, Sydney, NSW, Australia.; 5 Centre for Kidney Research, The Children’s Hospital, Westmead, NSW, Australia.; 6 Department of Renal and Transplantation Medicine, Westmead Hospital, Sydney, NSW, Australia.; 7 College of Medicine and Public Health, Flinders University, Adelaide, SA, Australia.; 8 Robert Wood Johnson Medical School, Rutgers University, New Jersey, NJ.; 9 Bristol-Myers Squibb Children’s Hospital, New Jersey, NJ.; 10 Department of Nephrology, Queensland Children’s Hospital, Brisbane, QLD, Australia.; 11 Faculty of Health, Medicine & Behavioural Sciences, The University of Queensland, Brisbane, QLD, Australia.; 12 Flinders Health and Medical Research Institute, College of Medicine and Public Health, Flinders University, Adelaide, SA, Australia.; 13 Renal Unit, Flinders Medical Centre, Adelaide, SA, Australia.; 14 Australasian Kidney Trials Network, The University of Queensland, Brisbane, QLD, Australia.; 15 Leeder Centre for Health Policy Economics and Data, University of Sydney, Sydney, NSW, Australia.; 16 Department of Nephrology, Monash Health, Melbourne, VIC, Australia.; 17 Department of Medicine, Centre for Inflammatory Diseases, Monash University, Melbourne, VIC, Australia.; 18 Department of Nephrology, Prince of Wales Hospital, Randwick, NSW, Australia.; 19 Centre for Kidney Research, The Children’s Hospital at Westmead, Sydney, NSW, Australia.; 20 Centre for Infectious Diseases and Microbiology Laboratory Services, Institute of Clinical Pathology and Medical Research, Westmead Hospital, Sydney, NSW, Australia.; 21 Department of Microbiology, Immunology and Transplantation, University of Leuven, Leuven, Belgium.; 22 Department of Nephrology and Renal Transplantation, University Hospitals Leuven, Leuven, Belgium.; 23 Department of Nephrology and Hypertension, Perth Children’s Hospital, Perth, WA, Australia.; 24 Medical School, University of Western Australia, Nedlands, WA, Australia.; 25 Nutrition and Health Innovation Research Institute, Edith Cowan University, Joondalup, WA, Australia.; 26 Division of Nephrology, Department of Medicine, University of Saskatchewan, Saskatoon, SK, Canada.; 27 Centre for Child Health Research, Faculty of Medicine, University of Western Australia, Perth, WA, Australia.; 28 Auckland City Hospital, Auckland, New Zealand.; 29 University of Auckland, Auckland, New Zealand.; 30 Sydney School of Public Health, Faculty of Medicine and Health, University of Sydney, Sydney, NSW, Australia.; 31 Prince of Wales Hospital, Sydney, NSW, Australia.; 32 Prince of Wales Clinical School, University of New South Wales Sydney, Sydney, NSW, Australia.; 33 Centre for Transplant and Renal Research, Westmead Hospital, Sydney, SA, Australia.; Royal Prince Alfred Hospital, The University of Sydney; Flinders Medical Centre, Flinders University; Canberra Hospital; The University of Queensland; Royal Prince Alfred Hospital; Monash Medical Centre, Monash University; John Hunter Hospital, The University of Sydney; John Hunter Hospital, University of New Castle; Monash Medical Centre, Monash University; Perth Children’s Hospital; Canberra Health Services, Australian National University; John Hunter Hospital, The University of Sydney

## Abstract

**Background.:**

BK polyomavirus (BKPyV) is a common opportunistic infection in kidney transplant recipients, typically reactivating in the context of immunosuppression. Although asymptomatic in immunocompetent individuals, reactivation in transplant recipients can cause BKPyV-associated nephropathy (BKPyVAN), a leading cause of graft dysfunction and loss. BKPyV viremia affects approximately 10%–15% of transplant recipients, and once BKPyVAN is established, the risk of graft failure can exceed 50%. The current standard of care involves immunosuppression reduction to restore virus-specific immunity, but this approach increases the risk of acute and chronic rejection. Intravenous immunoglobulin (IVIg), which contains antiviral and immunomodulatory antibodies, has been proposed as an adjunctive therapy, although evidence supporting its efficacy remains limited to small observational studies.

**Methods.:**

The BEAT-BK trial is a multicenter, adaptive, randomized controlled trial comparing standard immunosuppression reduction with or without IVIg in kidney and simultaneous pancreas-kidney transplant recipients with BKPyV viremia or biopsy-confirmed BKPyVAN. The trial will enroll participants aged 2 y and older, randomizing them 1:1 to receive standard care alone or in combination with a total IVIg dose of 2 g/kg for 8 wk. An adaptive Bayesian design will permit interim analyses with the potential for early trial success or futility.

**Results.:**

The primary endpoint is a composite ordinal outcome at 12 wk, incorporating death, graft loss, estimated glomerular filtration rate decline, acute rejection, viral load, and degree of immunosuppression reduction. Secondary outcomes include viral clearance, development of BKPyVAN and donor-specific antibodies, infections, malignancy, quality of life, and cost-effectiveness.

**Conclusions.:**

This trial will provide the first high-quality evidence on the role of IVIg in BKPyV management and inform clinical decision-making in posttransplant care.

## INTRODUCTION

BK polyomavirus (BKPyV) infection is a rare but devastating complication in kidney and simultaneous pancreas-kidney (SPK) transplant recipients. The overall incidence of BKPyV DNA in the blood (BKPyV-DNAemia) is approximately 10%–15%, with polyomavirus-associated nephropathy (BKPyVAN) occurring in approximately 5% of recipients within the first 12 mo posttransplant.^1,2^ BKPyV is a ubiquitous infection, typically acquired in childhood,which establishes latency in the native epithelial cells.^3^ Reactivation and progression to BKPyVAN occur exclusively in immunocompromised individuals.^4^ BKPyV DNAemia, detected by real-time polymerase chain reaction and leading to kidney dysfunction and BKPyVAN, is the fourth leading cause of graft loss among kidney and SPK transplant recipients worldwide. Once established, BKPyVAN carries a >50% risk of graft loss within 5 y.^5^

The strongest risk factor for BKPyV reactivation and disease is over-immunosuppression, particularly in the early posttransplant period. Other risk factors include the use of T-cell depleting agents, increased exposure to tacrolimus, older recipient or donor age, and male sex.^2,6^ Currently, apart from judicious reduction of immunosuppression, no effective interventions are available for treating BKPyV infections. Although adjuvant therapies are not commonly used, rescue treatments such as intravenous immunoglobulin (IVIg) have been used in patients with uncontrolled viraemia and ongoing graft deterioration when conventional immunosuppression reduction has failed. IVIg is a nondepleting agent containing natural antibodies with potential antiviral and immunomodulatory properties.^7^ It is used against some chronic infections (Epstein-Barr virus) and for the treatment of antibody-mediated rejection in kidney transplantation.^8^ However, in BKPyV infection, the certainty of the evidence for IVIg is very low because of imprecision and a high risk of bias (small, case series, retrospective cohorts). Still, it holds promise based on findings from observational data. In a single center, cohort study, recipients with BKPyV DNAemia who received IVIg as adjuvant therapy were more likely to achieve complete viral clearance at 12 mo (77.3% versus 33.3%, *P* < 0.01) and less likely to relapse (11% versus 27.3%, *P* = 0.01) compared with recipients who received conventional therapy alone.^9^ Therefore, high-quality trial-based evidence is needed to determine whether IVIg provides meaningful patient-relevant and clinically beneficial effects over standard care in kidney and SPK transplant recipients with BKPyV infections.

The BEAT-BK trial will evaluate the efficacy of immunosuppression reduction strategies, with and without the addition of IVIg, in kidney and SPK transplant recipients with BKPyV infection. The trial will assess the effects of these strategies on key clinical outcomes, including the severity of BKPyV infection, kidney allograft function, allograft loss (kidney and/or pancreas), acute transplant rejection, the degree of immunosuppression reduction required, and mortality.

### Protocol Design and Methods

This study is a multicenter, adaptive, 2-arm randomized trial designed to compare standard-of-care immunosuppression reduction or modification, with and without the addition of IVIg, in kidney and SPK transplant recipients with BKPyV infection. The trial is structured to assess multiple outcomes in parallel, including viraemia, acute rejection, immunosuppression load, allograft function and adverse events (AEs). The trial will employ an adaptive design with prespecified interim analyses, allowing for the potential to stop for early success or futility. The trial will use a Bayesian hierarchical modeling strategy to facilitate the empirical borrowing of information across related subgroups. This approach enables more efficient estimation of subgroup-specific intervention effects.^10^

### Study Setting

Patients will be recruited from kidney transplant units across Australia as well as from international sites including New Zealand, Canada, and the United States.

### Eligibility Criteria

To be eligible for trial inclusion, the patient must meet all of the following criteria:

1. Aged 2 y or older.
2. Received a kidney or SPK transplant.
3. Have BKPyV DNAemia, confirmed by real-time polymerase chain reaction calibrated to the World Health Organization international standard, with a viral load ≥ 5 × 10^3^ copies/mL (or equivalent 3.7 log_10_ copies/mL), or histologically confirmed BKPyVAN, within 3 wk before randomization.
4. Be legally capable of providing informed consent, or have consent provided by a parent, guardian, or other legally authorized representative (for patients younger than 18 y).

To be eligible, the patient must not meet any of the following criteria:

1. Presence of contraindications to IVIg treatment.
2. Current active acute rejection or rejection within the past 3 mo.
3. Considered by the treating clinician to be unsafe to enroll.
4. Estimated life expectancy of <12 mo.
5. Receiving belatacept as part of their immunosuppression regimen.
6. Currently receiving, or previously having received, virus-specific T-cell infusions for BKPyV infection.
7. History of prior IVIg therapy for BKPyV DNAemia during the current transplant.
8. IVIg therapy for any indication within 4 wk before randomization.

### Consent

Initial ethics approval for this trial was granted by Metro South Health Research, Queensland Government, Australia (HREC/2022/QMS/87487). Informed consent will be obtained before the initiation of any trial procedures, following approval of site-specific consent forms by the appropriate independent ethics committee. For pediatric participants, consent will be obtained from a parent or guardian. Where appropriate, children will also be provided with age-appropriate information and invited to cosign the consent form if judged sufficiently mature.

### Intervention

Participants will be randomized to 1 of 2 treatment arms: standard immunosuppression reduction alone or immunosuppression reduction in combination with IVIg. The total intended IVIg dose over the 8-wk intervention period will be 2 g/kg, with a tolerance range of 1.8–2.2 g/kg. The recommended dosing schedule is 250 mg/kg weekly for 8 wk, with a 2-wk administration window for flexibility. The first dose of IVIg must be given within 14 d of randomization. Because of potential supply and logistical constraints, the protocol permits IVIg doses to be combined, allowing for up to 2 doses (up to 500 mg/kg) to be administered during a single session.

In both arms, immunosuppression reduction is implemented per current standard of care following a standardized algorithm adapted from the 2024 Transplantation Society guidelines (Table 1).^11^ This typically involves a stepwise reduction in calcineurin inhibitors (CNIs) and antiproliferative agents, or conversion to less potent agents. The protocol provides flexibility for clinicians to alter immunosuppressive therapy according to local standards and clinical judgment. The treatment goal is to achieve a BK viral load <1000 copies/mL, where feasible.

**TABLE 1.** Recommended approach to immunosuppression reduction (adapted from The Second International Consensus Guidelines on the Management of BK Polyomavirus in Kidney Transplantation, 2024^11^)

Strategy 1: Antimetabolite is reduced first
Reduction of the dose of antimetabolite by at least 50%
We suggest further immunosuppression reduction if BKPyV DNAemia does not decrease by 10-fold at 4 wk or does not clear below lower limit of detection as follows:
Discontinuation of the antimetabolite and tapering of corticosteroid dose to 5–10 mg/d of prednisone or equivalent, if applicable
We suggest adding prednisone (or equivalent) 5–10 mg/d for patients who are not on corticosteroids to avoid CNI monotherapy
If further decrease in immunosuppression is necessary, we suggest a stepwise reduction of the CNI dose (tacrolimus trough target 5 ng/mL; cyclosporine trough target 100 ng/mL)
Strategy 2: CNI is reduced first
Reduction of the dose of CNI by 25%–50% in 1 or 2 steps to target trough concentrations of tacrolimus of 3–5 ng/mL and cyclosporine trough concentrations of 75–125 ng/mL)
We suggest further immunosuppression reduction if BKPyV DNAemia does not decrease by 10-fold at 4 wk or does not clear below lower limit of detection as follows:
Reduction of the antimetabolite by 50% and tapering of corticosteroid dose to 5–10 mg/d of prednisone or equivalent, if applicable
Discontinuation of the antimetabolite
We suggest adding prednisone (or equivalent) 5–10 mg/d for patients who are not on corticosteroids to avoid CNI monotherapy

BKPyV, BK polyomavirus; CNI, calcineurin inhibitor.

### Randomization and Blinding

Participants will be randomized in a 1:1 ratio using a secure, web-based randomization system embedded within the REDCap trial database. Randomization will be stratified by age, country, and transplant type (kidney alone or SPK), with the use of random permuted blocks to ensure balanced allocation across treatment groups within each center.

Given the nature of the intervention, complete blinding is not feasible. Therefore, clinicians and participants will be unblinded to the treatment allocation. However, measures are in place to preserve blinding among other key stakeholders. The BEAT-BK Global Steering Committee (GSC), site investigators, and the Trial Management Committee will remain blinded to individual treatment allocations and any aggregate data that could compromise unblinding of study outcomes. Although the statistical analysis team will be unblinded during interim analyses, a designated trial statistician will remain blinded throughout to support final primary analysis. All enrolled participants will be followed for a total of 48 wk after randomization.

### Primary Outcome

**FIGURE 1.**
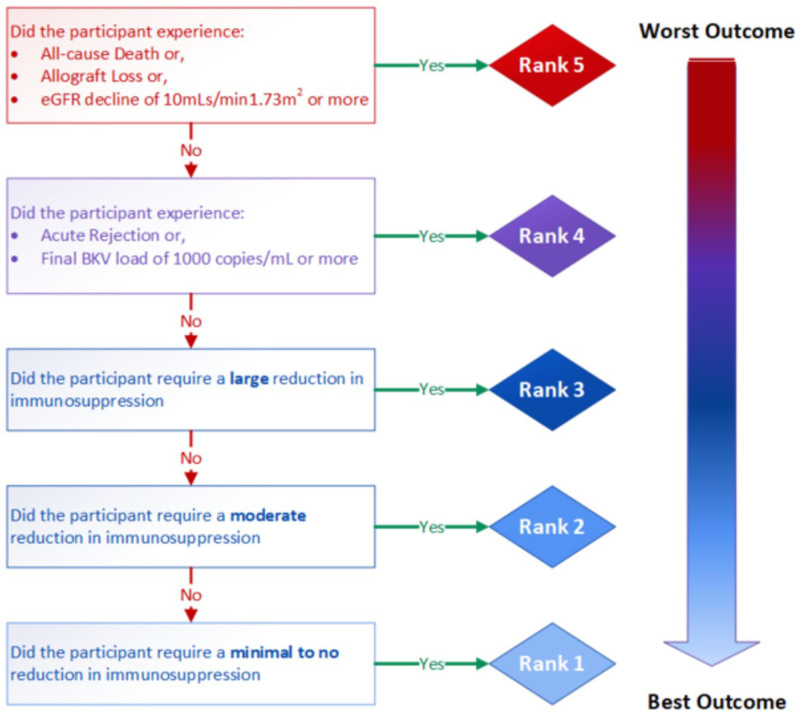
Primary composite ordinal outcome assessed at 12 wk.

The primary outcome is a composite, ordinal outcome assessed at 12 wk (with an acceptable window of 11–13 wk) after randomization (Table 2; Figure 1). Each participant will be assigned to a category on a 5-point ordinal scale, with higher scores indicating worse outcomes:

**TABLE 2.** Primary composite ordinal outcome at 12 wk postrandomization

Primary objective	Outcome measure(s)	Time-point(s)
To compare the efficacy of immunosuppression reduction/ modification strategies, with and without IVIg, on BKPyV infection in kidney and simultaneous kidney-pancreas transplantation recipients	A composite, ordinal outcome defined as: 5: All-cause death or allograft loss (kidney and/or pancreas) or eGFR decline ≥10 mL/min/1.73 m^2^ using the CKD-EPI formula^*a*^ for adults and the Schwartz formula^*a*^ for children 4: Acute rejection (kidney and/or pancreas) or BKV load >1000 copies/mL 3: Large reduction in immunosuppression relative to baseline 2: Moderate reduction in immunosuppression relative to baseline 1: Small to no reduction in immunosuppression relative to baseline Participants will be assigned a category between 1 (best) and 5 (worst).	Week 12

^*a*^ Pancreas graft function loss is defined as kidney-pancreas transplantion recipients’ insulin use is ≥0.5 units/kg/d for 90 consecutive days, or when the recipient’s pancreas graft is removed, and/ or re-registered for a pancreas transplantation.

BKPyV, BK polyomavirus; CKD-EPI, Chronic Kidney Disease Epidemiology Collaboration; eGFR, estimated glomerular filtration rate; IVIg, intravenous immunoglobulin; NB, all timepoints in the above table commence from the date of randomization.

Category 5: All-cause death *or* allograft loss (kidney and/or pancreas) *or* an estimated glomerular filtration rate (eGFR) decline of ≥10mL/min/1.73 m2 using the race-free creatinine-based Chronic Kidney Disease Epidemiology Collaboration equation formula for adults^12^ and the Chronic Kidney Disease in Children 2012 for children.^13^

Category 4: Biopsy-proven acute rejection (T cell–mediated, antibody-mediated, mixed, and borderline rejection in the kidney and/or pancreas) *or* has a BKPyV DNAemia exceeding 1000 copies/mL.

Category 3: Required a “large” reduction in immunosuppression relative to *baseline.

Category 2: Required a “moderate” reduction in immunosuppression relative to baseline.

Category 1: Required a “small” to no reduction in immunosuppression relative to baseline.

*Baseline is defined as “at the time of randomization.”

This composite ordinal outcome was chosen because BKPyV infection has multifaceted effects on both the allograft and the transplant recipient.^14^ BKPyV viral load is a reliable marker of infection, and at a threshold of 5 × 10^3^ copies/mL, has a positive predictive value of approximately 50% for BKPyVAN in kidney and SPK transplant recipients.^15,16^ Kidney allograft function, as measured by changes in eGFR, is known to predict longer-term allograft loss.^17^ Furthermore, a minimal requirement for immunosuppression reduction during BKPyV management likely reflects effective viral control and is associated with more favorable long-term outcomes.

Despite the utility of these measures, relying on any single outcome would be insufficient to capture the complex trade-offs involved in managing BKPyV.^14^ For instance, an aggressive reduction in immunosuppression may lead to a rapid decline in viral load, but at the cost of increased risk of acute rejection, graft dysfunction or even graft loss. Conversely, overly conservative management may preserve allograft function in the short term while allowing progressive viral injury to develop. To address these competing risks, the ordinal composite outcome has been adopted. Participants will be categorized based on a combination of death, graft loss, rejection, graft dysfunction, viral load, and the degree of immunosuppression reduction required, providing a more holistic assessment of treatment response.

Reductions in immunosuppression will be defined as minimal, moderate, or large, depending on the degree of immunosuppression reduction required at 12 wk postrandomization (Table 3). The largest reduction in any single immunosuppressive medication (excluding steroid therapy) will be considered for outcome classification. Reductions in antimetabolites will be categorized according to the percentage reduction relative to initial dosing. Reductions in tacrolimus, ciclosporin, and mammalian target of rapamycin (mTOR) inhibitors will be categorized as the reduction in trough target levels at 12 wk relative to initial target trough levels. For patients receiving tacrolimus, ciclosporin, and mTOR inhibitors, their treating clinicians will be asked to specify target levels at randomization and again at 12 wk. Reductions in steroid dosing will not be considered part of the ranked outcome. Substantial variation exists between centers in the use of steroids after transplantation, with some centers adopting steroid-free programs. Therefore, in this cohort, reductions in steroid doses may be unrelated to BKPyV infections.

**TABLE 3.** Classification of immunosuppression reduction by degree of reduction

Immunosuppression medication	Degree of reduction^*a*^	Large	Moderate	Minimal
Antimetabolite	Dose reduction from baseline (%)	≥75%	>25–<75%	≤25%
Ciclosporin (C0 or C2)	Target-level reduction from baseline (%)	≥75%	>25–<75%	≤25%
mTOR-I (trough)				
Tacrolimus (trough, ng/mL)	Baseline trough	Final target trough	Final target trough	Final target trough
	≥8	≤3.9 (or cessation)	4–6.9	≥7
	6–8	≤3.9 (or cessation)	4–5.9	≥6
	4–6	≤2.9 (or cessation)	3–3.9	≥4
	<4	Cessation	≤2.9 (>0)	≥3

^*a*^ The largest reduction in any single immunosuppressive medication (excluding steroid therapy) will be considered for outcome classification.

mTOR, mammalian target of rapamycin.

In addition to dose reductions, conversion from a more potent to a less potent immunosuppressive agent in direct response to BKPyV infection will be classified as a “large” reduction. Conversions performed for other indications, such as drug intolerance, adverse effects, or malignancy, should not be considered a “large” reduction.

Conversions that qualify as “large” immunosuppressive reductions include:

- Tacrolimus to ciclosporin;
- Tacrolimus to an mTOR inhibitor;
- Ciclosporin to an mTOR inhibitor;
- Conversion to from a CNI to azathioprine alone;
- Mycophenolate mofetil/mycophenolic acid to azathioprine;
- Mycophenolate mofetil/mycophenolic acid to an mTOR inhibitor;
- Azathioprine to an mTOR inhibitor.

### Secondary Outcomes

The secondary outcomes for this trial were selected in alignment with internationally endorsed transplant outcome standards, including the Standardized Outcomes in Nephrology–Kidney Transplantation^18^ core domains (Table 4). These outcomes will be evaluated at 12-, 24-, and 48-wk postrandomization and encompass indicators of virological response (BKPyV DNAemia, BKPyVAN), graft function (decline in kidney function, allograft loss, biopsy-proven acute rejection), immunological activity (development of de novo donor-specific antibodies), and clinical events (death, hospitalizations, venous thromboembolism events, cancer diagnoses). To account for changes in immunosuppression that may occur before randomization, a sensitivity analysis will evaluate the primary composite ordinal outcome using the immunosuppressive regimen in place at the time the patient first recorded a viral load >5000 copies/ml, rather than the regimen at randomization. This analysis will help determine whether prerandomization modifications influenced the primary outcome.

**TABLE 4.** Secondary outcomes

Secondary objectives	Outcome measure(s)	Time-point(s)
To assess the proportion of participants with a BKPyV viral load reduction between the intervention and control groups	BKPyV final viral load reduction of ≥3 log_10_ or <1000 copies/mL	Week 12
To assess graft function in participants with BKPyV between the intervention and control groups	eGFR decline ≥10 mL/min/1.73 m^2^	Weeks 12, 24, 48
To compare mortality in participants with BKPyV between the intervention and control groups	All-cause death	Weeks 12, 24, 48
To compare graft survival and death-censored graft survival in participants with BKPyV between the intervention and control groups	Graft loss	Weeks 12, 24, 48
To compare the number of acute rejections (cellular- and antibody-mediated) episodes in participants with BKPyV between the intervention and control groups	Acute rejection of kidney and/or pancreas allografts	Weeks 12, 48
To compare the development of de novo donor-specific antibodies in participants with BKPyV between the intervention and control groups	Donor-specific anti-HLA antibody	Weeks 12, 48
To compare the incidence of venous thromboembolism in participants with BKPyV between the intervention and control groups	Venous thromboembolism events	Week 12
To compare the number of hospitalizations because of infection in participants with BKPyV between the intervention and control groups	Hospitalizations because of infection events	All time points
To compare infectious events requiring antimicrobial therapy in participants with BKPyV between the intervention and control groups	Infectious events requiring antimicrobial (antibacterial, antiviral, antifungal, antiprotozoal) therapy	All time points
To compare health-related quality of life in participants with BKPyV between the intervention and control groups	EQ-5D-5L for adults/HUI-3 for children	Baseline, weeks 12, 24, 48
To compare the development of BKPyVAN in participants with BKPyV between the intervention and control groups	BKPyVAN events	Weeks 12, 48
To compare cancer outcomes in participants with BKPyV between the intervention and control groups	Any cancer diagnosis or cancer-related death	Weeks 24, 48
To assess the long-term composite ranked outcome in participants with BKPyV between the intervention and control groups	Composite ordinal outcome (see primary outcome)	Week 48
To assess the cost-effectiveness of IVIg therapy compared with current practice in participants with BKPyV	Incremental cost-effectiveness ratios for the primary outcomes, and quality adjusted life years	Week 48
To assess long-term graft and patient outcomes in participants enrolled from Australia and New Zealand	Linked registry data from the Australia and New Zealand Dialysis and Transplant Registry, including graft function, rejection, graft loss, and death	Posttrial follow-up (via data linkage)
To evaluate the impact of prerandomization immunosuppression changes on the primary composite ordinal outcome using the immunosuppressive regimen in place at the time of first qualifying BKPyV DNAemia (>5000 copies/mL)	Primary composite, ordinal outcome	Weeks 12, 48

BKPyV, BK polyomavirus; BKPyVAN, BK polyomavirus-associated nephropathy; eGFR, estimated glomerular filtration rate; EQ-5D-5L, EuroQol 5-dimension 5-level; HUI-3, health utilities index mark 3; IVIg, intravenous immunoglobulin.

In addition, quality of life will be assessed using validated, age-appropriate instruments, and longer-term effects of the intervention will be captured through a composite ordinal outcome at 48 wk. A trial-based economic evaluation, including healthcare resource utilization and cost-effectiveness of IVIg, will also form part of the secondary analysis.

For participants enrolled in Australia and New Zealand, data linkage with the Australia and New Zealand Dialysis and Transplant Registry will enable extended follow-up beyond 48 wk. Linked outcomes that will include graft function, graft loss, rejection episodes, and death. This longer-term follow-up will facilitate evaluation of the delayed or sustained effects of the intervention that may not be apparent within the initial trial observation period.

### Statistical Analyses

The objective of the primary estimand is to compare the efficacy of immunosuppression reduction with and without IVIg using a composite ordinal outcome at 12 wk postrandomization. This outcome includes 5 levels, ranging from the most favorable (level 1: minimal immunosuppression reduction) to the most severe (level 5: death, allograft loss, or significant decline in kidney function). Anticipated intercurrent events that may occur post-randomization and either preclude or affect the outcome include nonadherence to allocated intervention (because of site procedures, availability, and clinical indication), loss to follow-up or withdrawal, missing histopathology/biopsy results or receipt of a new kidney transplant. For each of these events a “treatment-policy” strategy will be employed where available data will be used up to the time of the event (eg, if a participant is lost to follow-up before 12 wk, then their available data will be used to inform their 12-wk outcome).^19^ **Table S1 (SDC,** https://links.lww.com/TXD/A839) details the estimands (including for the secondary outcomes) and associated intercurrent event strategies.

All statistical analyses will be stratified by graft type (kidney or simultaneous pancreas and kidney) and age group (<60 or ≥60 y) and adjust for site and other relevant covariates (eg, sex). Continuous covariates will be standardized within stratum (graft type/age group combination), and the reference value for categorical covariates will be set to the most frequently observed category. The analysis for the primary estimand will use a Bayesian proportional odds cumulative logistic regression model. The treatment effect (intervention compared with standard of care) will be reported as a posterior distribution of the common odds ratio of a higher ordinal category (ie, worse outcome). The reference stratum will be the combination of a kidney graft type and the <60 y age group. Model parameters will have weakly informative prior distributions including standard Gaussian distributions for the stratum, intervention and covariate parameters, and a Dirichlet distribution (concentration of 1) for the cut points.^20^

An exploratory model will assess treatment effect heterogeneity (across graft type and age group strata) with a stratum-specific treatment effect parameter and a 2-level hierarchical structure that allows for data-driven information borrowing across strata.^10^ The exploratory model estimates the common odds ratio for the intervention compared with standard of care in each stratum. Models for the secondary estimands include a linear model (for continuous outcomes, eg, change in eGFR), logistic model (for binary outcomes, eg, development of De novo donor-specific antibodies) and a Weibull survival model (for time to event outcomes, eg, time of allograft loss). The linear, logistic and survival models estimate the posterior distributions of the linear (additive) effect, odds ratio and hazard, respectively, for the intervention compared with standard of care. Weakly informative prior distributions will be used for all model parameters to support estimation while allowing flexibility.

The adaptive trial design includes prespecified interim analyses occurring after 100 participants reach the primary endpoint, and every 60 thereafter. Trial adaptations are based on the posterior probability that the common odds ratio from the primary estimand is <0.9 (ie, the common odds ratio that a participant allocated to the intervention arm will have a better ordinal outcome than a participant allocated to the standard-of-care arm). The trial will stop early for superiority or futility if this posterior probability exceeds 95% or falls below 10%, respectively.

Extensive simulations were conducted to inform key design parameters, including the maximum sample size (n = 280). These simulations evaluated the various scenarios of the treatment effects, the event rates, interim analysis timing, and intercurrent events. For each scenario, 5000 trials were simulated assuming 5% loss to follow-up by 12 wk after randomization. The modeling confirmed that the trial design maintains robust control of type 1 error while achieving adequate statistical power to detect clinically relevant treatment effects across a range of plausible assumptions.

All summary statistics, analyses, and data visualizations will be generated in R version 4.2.2 or later.^21^ A detailed description of the trial estimands, statistical models, decision criteria for adaptations and justification of the trial design by simulation are outlined in **Supplementary File 1** (**SDC**, https://links.lww.com/TXD/A839), with simulation results presented in **Figure S1 and Table S2** (**SDC**, https://links.lww.com/TXD/A839).

### Trial Oversight

The study will be conducted and coordinated by the Australasian Kidney Trials Network. The study will be conducted according to Good Clinical Practice guidelines and the reporting of results will follow the Consolidated Standards of Reporting Trials guidelines.^22^

The Australasian Kidney Trials Network will establish and manage key oversight bodies, including an independent Data and Safety Monitoring Board (DSMB), an Endpoint Verification Committee, and a Consumer Advisory Board. The DSMB, comprising independent experts in clinical trials, nephrology, infectious diseases, and biostatistics, will monitor participant safety and trial progress, and may recommend early termination because of efficacy or safety concerns. While the DSMB will report to the GSC Chair, all final decisions on trial modifications or early stopping will rest with the GSC.

### Safety

Participant safety will be closely monitored throughout the trial, with all AEs, adverse drug reactions, serious AEs, suspected unexpected serious adverse reactions, and AEs of special interest reported by site staff and reviewed by the DSMB. Investigators will assess the relationship between any adverse event and IVIg administration using a standardized scale (none, unlikely, possible, or probable). All safety data will be reviewed by the DSMB (**Table S2, SDC,** https://links.lww.com/TXD/A839), which is responsible for evaluating participant safety and determining whether any trial modifications or early stopping are warranted.

### Data Security, Confidentiality, and Handling

All data collected in the BEAT-BK study will be stored securely in accordance with national regulations and the data management procedures outlined in the Country-Specific Appendices. Identifiable information will remain confidential and accessible only to the research team. Article-based documents will be stored in a locked facility, while electronic data will be maintained on a secure, access-restricted online platform. Personal information will be used solely for the study. Consent forms will be retained locally, and all study-related documentation, including ethics approvals and correspondence, will be archived for up to 15 y after trial closure or until the youngest participant reaches 25 y of age, whichever is later. Datasets will be made available to BEAT-BK investigators for approved substudies once the primary article is published. For external researchers, de-identified individual participant data may be accessed upon request to a dedicated Data Access Committee.

## DISCUSSION

The BEAT-BK trial addresses a key unmet need in kidney and SPK transplant recipients, as BKPyV infection is a clinically significant complication that can result in progressive graft dysfunction and loss. Immunosuppression reduction aims to restore BKPyV-specific cellular immunity, thereby facilitating viral clearance. However, this approach is not without risks. Inadequate immunosuppression may result in both acute and chronic rejection, leading to irreversible graft injury and potential graft loss. IVIg has been proposed as a potential therapeutic candidate because of its immunomodulatory and antiviral properties. Observational evidence suggested that IVIg has been associated with a rise in genotype-specific neutralizing antibody responses to BKPyV infections and, therefore, a reduced risk of BKPyVAN in high-risk populations. However, the lack of high-quality evidence has precluded definitive conclusions regarding the clinical efficacy of this treatment in treating BKPyV infection in transplant recipients. Also, IVIg is associated with known risk, including hypersensitivity reactions, thromboembolic events, acute kidney injury, and fluid overload, and carries a significant financial cost.

Using a novel Bayesian adaptive design, the trial framework enables planned interim analyses and sample size re-estimation, facilitating timely decisions regarding trial continuation or early stopping for efficacy or futility. More importantly, it enables efficient resource allocation, directing efforts toward the most promising treatment strategies and exposing fewer patients to inferior treatments. The selection of a 12-wk endpoint reflects a clinically meaningful timeframe for assessing early viral clearance, immunological and graft-related outcomes. In the absence of approved and effective antiviral treatments for BKPyV infection, the findings of the BEAT-BK trial will provide critical evidence to guide therapeutic decision-making. If found to be effective, IVIg could offer a viable strategy to improve long-term graft outcomes in kidney and SPK transplant recipients. If IVIg is deemed ineffective, the trial findings will then support de-implementation of an expensive and ineffective treatment, thereby avoiding unnecessary exposure to potential harms.

## ACKNOWLEDGMENTS

The authors acknowledge the contributions of the following individuals to the BEAT-BK and this article: Mia E Abdy (The Children’s Hospital at Westmead), Rita Barbis (Monash Medical Centre), David Charman (The University of Queensland), Ross S Francis (Princess Alexandra Hospital), Rachael Hale (Princess Alexandra Hospital), Peter Hughes (The Royal Melbourne Hospital), Rabia Khalid (The Children’s Hospital Westmead), Merin Kuriakose (Flinders Medical Centre), Diana R Leary (Princess Alexandra Hospital), Lizanne Mackintosh (Canberra Hospital), Susan Maddocks (Westmead Hospital), Misa Matsuyama (The University of Queensland), Ramya Movva (The University of Queensland), Melissa Mulholland (John Hunter Hospital), Peta-Anne Paul-Brent (The University of Queensland), Donna Reidlinger (The University of Queensland), Helio Tedesco silva Junior (University of Sao Paulo), Fleur Tuthill (Flinders Medical Centre), Julie Varghese (The University of Queensland), Pushparaj Velayudham (The University of Queensland), Rosemary G Widdison (John Hunter Hospital).

Collaborators & BEAT-BK investigators: David Gracey (Royal Prince Alfred Hospital, The University of Sydney), Rajiv Juneja (Flinders Medical Centre, Flinders University), Krishna Karpe (Canberra Hospital), Charani Kiriwandeniya (The University of Queensland), Lin Lin (Royal Prince Alfred Hospital), William R Mulley (Monash Medical Centre, Monash University), Thida Maung Myint (John Hunter Hospital, The University of Sydney), Matthew Rolandson (John Hunter Hospital, University of New Castle), Jessica Ryan (Monash Medical Centre, Monash University), Dawn Sheriff (Perth Children’s Hospital), Girish Talaulikar (Canberra Health Services, Australian National University), Eswari Vilayur (John Hunter Hospital, The University of Sydney)

## Supplementary Material


