# Supplementary material for: BEAT-BK: An Adaptive, Randomized Controlled Trial to Treat Polyomavirus Infections (BKPyV) in Kidney and Kidney-pancreas Transplantation Recipients (BEAT-BK) Study Protocol
Source: Transplant Direct. 2026 Mar 4;12(4):e1924. doi: 10.1097/TXD.0000000000001924 (PMC12962577; doi:10.1097/TXD.0000000000001924)
Supplement: Supplementary file 1 [file txd-12-e1924-s001.pdf]

BEAT-BK: An adaptive, randomized controlled trial to treat polyomavirus infections (BKPyV) in kidney and kidney-pancreas transplant recipients (BEAT-BK) – study protocol

Supplementary File 1

Contents

OUTLINE **2**

TRIAL STRUCTURE **3**

PARTICIPANTS 3
GRAFT TYPE AND AGE GROUP (STRATA) 3
INTERVENTIONS 3
COVARIATES 3

ESTIMANDS **4**

STATISTICAL MODELLING **10**

DEMOGRAPHICS AND BASELINE CHARACTERISTICS 10
PRIMARY MODEL 10
CONTINUOUS ENDPOINT MODEL 11
BINARY ENDPOINT MODEL 11
TIME TO EVENT ENDPOINT MODEL 12
EXPLORATORY MODEL 12

STATISTICAL QUANTITIES **14**

QUANTITIES OF INTEREST 14
SUPERIORITY 14
FUTILITY 14

SEQUENTIAL ANALYSES (INTERIMS) AND TRIAL ADAPTATIONS **15**

FREQUENCY AND TIMING OF SEQUENTIAL ANALYSES (INTERIMS) 15
EVALUATION AND REPORTING OF DECISION CRITERIA 15

SIMULATIONS AND SAMPLE SIZE **16**

PREDEFINED ADVERSE EVENTS OF INTEREST ASSOCIATED WITH IVIG **19**

Outline

The focus of this supplementary file is to describe the trial estimands, statistical models, decision criteria for pre-specified adaptions and the trial simulations.

Trial Structure

Participants

We denote N as the maximum number of participants in any given analysis where participants are denoted $i \in \boldsymbol{I} = \{1,2,\dots,N\}$.

Graft type and age group (strata)

Graft type is denoted by $j \in \boldsymbol{J} = \{\text{Kidney [K], Simultaneous Pancreas and Kidney [SPK]}\}$ and age group is denoted by $l \in \boldsymbol{L} = \{< 60y, \geq 60y\}$. Four strata are defined as mutually exclusive groups based on participant graft type and age group.

Interventions

Participants are randomised with equal allocation probability to immunosuppression reduction/modification (standard of care) with and without IVIg. The trial arms are denoted by $k \in \boldsymbol{K} = \{1,2,\dots,K^*\}$ where 1 = Standard of care (without further interventions), denoted as *SoC,* and 2 = Standard of care with IVIg, denoted as *IVIg,* and K^* is the maximum number of arms. Note that the trial will start with two arms (SoC and IVIg) but has the flexibility to accommodate additional arms in the future.

Covariates

Participant i's covariates, including site, are denoted $\boldsymbol{\omega}_i = \{\omega_{i1}, \omega_{i2}, \dots, \omega_{iP}\}$ and are governed by P model parameters. Continuous covariates are standardised within stratum and the reference value for categorical covariates is set to the most frequently observed. The covariates may differ for the secondary models.

Estimands

The trial population includes participants that met the inclusion and exclusion criteria described in the manuscript and were randomized, hereafter known as eligible participants. The estimands, including the handling of common and/or anticipated intercurrent events, are defined in Table S1.

Table S1 BEAT-BK trial estimands. The standard of care and intervention arms are denoted SoC and IVIg, respectively. Eligible participants are those that met the inclusion and exclusion criteria and were randomized.

Estimand	Endpoint	Intercurrent events strategy
**Estimand 1**<br><br>To compare the efficacy of SoC and IVIg on a composite ordinal endpoint based on outcomes for BKPyV infection, allograft function, allograft loss, acute transplant rejection and death at 12 weeks after randomization in eligible participants.	**Endpoint:** Composite ordinal endpoint at 12 weeks* after randomization, assigned by clinically-qualified assessors blinded to treatment allocations and defined as:<br><br>5: All cause death *or* allograft loss (kidney and/or pancreas) *or* eGFR decline >10mls/min1.73m^2<br><br>4: Acute rejection (kidney and/or pancreas) or BKV load >1,000 copies/mL<br><br>3: Large reduction in immunosuppression<br><br>2: Moderate reduction in immunosuppression<br><br>1: Small to no reduction in immunosuppression<br><br>Category 1 represents the best response and 5 the worst.<br><br>**Population summary**: Proportional odds ratio of IVIg compared to SoC.	Anticipated intercurrent events that may occur post randomization and either preclude or affect the endpoint are:<br><br>(1) non-adherence to allocated intervention due to site procedures or availability.<br><br>(2) non-adherence to allocated intervention due to a serious adverse event or perceived clinical indication.<br><br>(3) loss to follow-up or withdrawal and unrelated to death.<br><br>(4) missing blood sample(s) at assessment timepoint, which is unrelated to death.<br><br>(5) where indicated, histopathology unavailable/biopsy not performed by assessment timepoint and unrelated to death.<br><br>(6) participant receives a kidney transplant or is admitted to ICU prior to time of endpoint: ‘treatment policy’ strategy, where only data collected prior to the transplant or

		transfer to ICU is used to infer the endpoints.<br><br>No modifications or corrections to the endpoint will be made due to non-adherence to allocated intervention, items (1) and (2) above, i.e., a treatment policy strategy. Participants lost to follow-up, withdrawn or missing blood samples or histopathology (each unrelated to death), items (3), (4) and (5) above, will have their data used from randomization up until the time of the endpoint or time of lost to follow-up/withdrawal/missing outcome, whichever is sooner, to infer the endpoint, i.e., a treatment policy strategy. Participants that receive a subsequent kidney transplant or are transferred to ICU, item (6) above, will have their data used from randomization up until the time of the endpoint or time of transplant/ICU transfer, whichever is sooner, to infer the endpoint, i.e., a treatment policy strategy.
**Estimand 2**<br><br>To compare the efficacy of SoC and IVIg on<br>the proportion of participants with a BKPyV viral load reduction of ≥3 $\log_{10}$ or absolute value <1,000 copies/mL at 12 weeks after	**Endpoint:** Categorised (binary) BKPyV viral load reduction of ≥3 $\log_{10}$ or absolute value <1,000 copies/mL at 12 weeks* after randomization.<br><br>**Population summary:** Odds ratio of IVIg compared to SoC.	As for Estimand 1 in addition to:<br><br>(7) death or graft loss that precludes the assessment of the endpoint will be included as a BkPyV viral load reduction of <3 $\log_{10}$ and >1,000 copies/mL, i.e., a composite strategy.

randomization in eligible participants.		
**Estimands 3-5**<br><br>To compare the efficacy of SoC and IVIg on the proportion of participants with an eGFR decline ≥10 ml/min/1.73m^2 at 12, 24, and 48 weeks after randomization in eligible participants.	**Endpoint:** Categorised (binary) eGFR decline ≥10 ml/min/1.73m^2 at 12 , 24, and 48 weeks* after randomization.<br><br>**Population summary:** Odds ratio of IVIg compared to SoC.	As for Estimand 1 in addition to:<br><br>(8) death or graft loss that precludes the assessment of the endpoint will be included as an eGFR decline ≥10 ml/min/1.73m^2, i.e., a composite strategy.
**Estimand 6**<br><br>To compare the efficacy of SoC and IVIg on mortality at 12, 24, and 48 weeks after randomization in eligible participants.	**Endpoint:** Time to death, censored at 48 weeks after randomization.<br><br>**Population summary:** Hazard ratio of IVIg compared to SoC and estimated proportion at 12, 24 and 48 weeks after randomization.	Items (1), (2), (3) and (6) from Estimand 1. Data will be censored at the time of loss to follow-up/withdrawal (3) or kidney transplantation/ICU transfer (6), i.e., a treatment policy strategy.
**Estimand 7**<br><br>To compare the efficacy of SoC and IVIg on graft survival at 12, 24 and 48 weeks after randomization in eligible participants.	**Endpoint:** Time to graft loss, censored at 48 weeks after randomization.<br><br>**Population summary:** Hazard ratio of IVIg compared to SoC and estimated proportion at 12, 24 and 48 weeks after randomization.	As for Estimand 6 in addition to:<br><br>(9) deaths that preclude the assessment of the endpoint will be included as a graft loss at the time of death, i.e., a composite strategy.
**Estimand 8**<br><br>To compare the efficacy of SoC and IVIg on the time of first acute rejection (cellular and antibody mediated) in eligible participants.	**Endpoint:** Time to first acute rejection censored at 48 weeks after randomization.<br><br>**Population summary:** Hazard ratio of IVIg compared to SoC.	As for Estimand 6 in addition to:<br><br>(10) death or graft loss that precludes the assessment of the endpoint will be included as an acute rejection at the time of death or graft loss, i.e., a composite strategy.

**Estimands 9-10**<br><br>To compare the efficacy of SoC and IVIg on acute rejection (cellular and antibody mediated) at 12 and 12–48 weeks after randomization in eligible participants.	**Endpoint:** Acute rejection (binary) at ≤12 weeks* and 13–48 weeks* after randomization.<br><br>**Population summary:** Odds ratio of IVIg compared to SoC.	As for Estimand 1 in addition to item (10).
**Estimand 11**<br><br>To compare the efficacy of SoC and IVIg on the time of development of de novo donor-specific antibodies in eligible participants.	**Endpoint:** Time to development of de novo donor-specific antibodies censored at 48 weeks after randomization.<br><br>**Population summary:** Hazard ratio of IVIg compared to SoC.	As for Estimand 6 in addition to:<br><br>(11) death or graft loss that precludes the assessment of the endpoint will be included as the development of de novo donor-specific antibodies at the time of death or graft loss, i.e., a composite strategy.
**Estimands 12-13**<br><br>To compare the efficacy of SoC and IVIg on the development of de novo donor-specific antibodies at 12 and 12-48 weeks after randomization in eligible participants.	**Endpoint:** Development of de novo donor-specific antibodies (binary) at ≤12 weeks* and 13–48 weeks* after randomization.<br><br>**Population summary:** Odds ratio of IVIg compared to SoC.	As for Estimand 1 in addition to item (11).
**Estimand 14**<br><br>To compare the efficacy of SoC and IVIg on venous thromboembolism events up to 12 weeks after randomization in eligible participants.	**Endpoint:** Venous thromboembolism events (binary) at ≤12 weeks* after randomization.<br><br>**Population summary:** Odds ratio of IVIg compared to SoC.	As for Estimand 1 in addition to:<br><br>(12) deaths that preclude the assessment of the endpoint will be included as a venous thromboembolism event, i.e., a composite strategy.
**Estimand 15**<br><br>To compare the efficacy of SoC and IVIg on first	**Endpoint:** Time to first hospitalisation due to infection	As for Estimand 6 in addition to:<br><br>(13) deaths that preclude the assessment of the endpoint will be

hospitalisation due to infection after randomization in eligible participants.	censored at 48 weeks after randomization.<br><br>**Population summary:** Hazard ratio of IVIg compared to SoC.	included as an infection at the time of death, i.e., a composite strategy.
**Estimand 16**<br><br>To compare the efficacy of SoC and IVIg on infectious events requiring antimicrobial (antibacterial, antiviral, antifungal, antiprotozoal) therapy up to 12 weeks after randomization in eligible participants.	**Endpoint:** Any infectious event requiring antimicrobial therapy (binary) at ≤12 weeks* after randomization.<br><br>**Population summary:** Odds ratio of IVIg compared to SoC.	As for Estimand 1 in addition to:<br><br>(14) deaths that preclude the assessment of the endpoint will be included as an infection requiring antimicrobial therapy, i.e., a composite strategy.
**Estimands 17-34**<br><br>To compare the efficacy of SoC and IVIg on health-related quality of life in participants at 12, 24, and 48 weeks after randomization in eligible adult* participants.<br><br>*Paediatric quality of life (HUI-3) will be summarised using median and interquartile range by intervention and stratum.	**Endpoint:** EQ-5D-5L total score and the five domain-specific scores at 12, 24, and 48 weeks* after randomization.<br><br>**Population summary:** Difference in scores between IVIg and SoC.	As for Estimand 1 in addition to:<br>(15) if death precludes the assessment of the EQ-5D-5L at any timepoint, domain-specific total scores of zero will be assigned, i.e., a composite strategy.
**Estimands 35-36**<br><br>To compare the efficacy of SoC and IVIg on the development of BKPyVAN in participants up to 12 weeks and up to 48 weeks after randomization in eligible participants.	**Endpoint:** Development of BKPyVAN (binary) at ≤12 weeks* and at ≤48 weeks* after randomization.<br><br>**Population summary:** Odds ratio of IVIg compared to SoC.	As for Estimand 1 in addition to:<br>(16) death or graft loss that precludes the assessment of the endpoint at the timepoint will be included as a development of BKPyVAN, i.e., a composite strategy.

**Estimand 37**<br><br>To compare the efficacy of SoC and IVIg on the time to cancer diagnosis after randomization in eligible participants.	**Endpoint:** Time to first cancer diagnosis censored at 48 weeks after randomization.<br><br>**Population summary:** Hazard ratio of IVIg compared to SoC.	As for Estimand 6 in addition to:<br><br>(17) death that precludes the assessment of the endpoint will be included as a cancer diagnosis at the time of death, i.e., a composite strategy.
**Estimand 38**<br><br>To compare the efficacy of SoC and IVIg on a composite ordinal endpoint based on outcomes for BKPyV infection, allograft function, allograft loss, acute transplant rejection and death at 48 weeks after randomization in eligible participants.	**Endpoint:** As for Estimand 1 at 48 weeks* after randomization.<br><br>**Population summary:** Proportional odds ratio of IVIg compared to SoC.	As for Estimand 1.
**Estimand 39**<br><br>To compare the efficacy of SoC and IVIg on the time to reaching rank 4 or 5 (as defined by the ordinal outcome) by 12 weeks after randomization in eligible participants.	**Endpoint:** Time to death or allograft loss or eGFR decline >10mls/min1.73m^2 or acute rejection (kidney and/or pancreas) or BKV load >1,000 copies/mL (i.e. primary endpoint categories 4 and 5) censored at 12 weeks* after randomization.<br><br>**Population summary**: Hazard ratio of IVIg compared to SoC.	As for Estimand 6.

* observations collected within ±1 week of the 12 week timepoints and ±2 weeks of the 24 and 48 week timepoints will be included.

Statistical Modelling

Demographics and baseline characteristics

Trial demographics and baseline characteristics will be summarised by stratum and intervention. Continuous variables with symmetric distributions will be summarised by mean and standard deviation, whereas those with asymmetric distributions will be summarised by median and interquartile range. Categorical variables will be summarised as frequency and percentage for each level.

Primary model

A Bayesian proportional odds cumulative logistic regression model will be used for the primary analysis (estimand 1) and estimand 38. The model estimates the posterior distribution of the common odds ratio of a higher ordinal outcome for the IVIg arm compared to the SoC arm measured 12 weeks after randomization (or 48 weeks for estimand 38). We denote the ordinal endpoint for individual i, with graft type *j*, allocated to intervention *k* and in age group *l* with $Y_{ijkl} \in \{1,2,3,4,5\}$. We denote ordered cut points $\boldsymbol{c} = \{c_1, c_2, c_3, c_4\}$ such that $c_1 < c_2 < c_3 < c_4$, and define the model:

$$Y_{ijkl} \sim \text{MultiNomial}(\text{n} = 1, [\pi_1, \pi_2, \pi_3, \pi_4, \pi_5]) \quad \forall i \in \boldsymbol{I}, j \in \boldsymbol{J}, k \in \boldsymbol{K}, l \in \boldsymbol{L}$$

$$\pi_t = \text{P}\left(Y_{ijkl} = t \middle| \eta_{ijkl}, \boldsymbol{c}\right) = \begin{cases} 1 - \text{logit}^{-1}\left(\eta_{ijkl} - c_1\right) & \text{if } t = 1 \\ \text{logit}^{-1}\left(\eta_{ijkl} - c_{t-1}\right) - \text{logit}^{-1}\left(\eta_{ijkl} - c_t\right) & \text{if } 2 \leq t \leq 4 \\ \text{logit}^{-1}\left(\eta_{ijkl} - c_4\right) & \text{if } t = 5 \end{cases}$$

$$\eta_{ijkl} = \alpha_{jl} + \beta_{k^*} + \sum_{p=1}^{P} \omega_{ip}\gamma_p \quad \forall i \in \boldsymbol{I}, j \in \boldsymbol{J}, k^* \in \{\boldsymbol{K} | k > 1\}, l \in \boldsymbol{L}$$

We set the parameter $\alpha_{j=\text{Kidney},l=\text{2y}-<\text{60y}} = 0$ so that the model is identifiable (i.e., the reference stratum is set to kidney alone graft type and age group 2y to <60y). We interpret α_{jl} as the common log-odds ratio that a participant in stratum $j \times l$ has a higher ordinal outcome compared to a participant in the reference stratum. The parameter of interest β_2 is then the common log-odds ratio that a participant allocated to the IVIg arm has a higher ordinal outcome than a participant allocated to the SoC arm. The common odds ratio for covariate p is then γ_p.

The weakly informative prior distributions are:

$$\alpha_{jl} \sim \mathrm{N}(0,1) \qquad \forall j \in \boldsymbol{J}, l \in \boldsymbol{L}$$

$$\beta_{k^*} \sim \mathrm{N}(0,1)\ \forall k^* \in \{\boldsymbol{K} | k > 1\},$$

$$\gamma_p \sim \mathrm{N}(0,1) \quad \forall p \in \{1,2,\dots,P\}$$

The cut points $\boldsymbol{c}$ are derived from a simplex and Dirichlet prior with concentration one.

Continuous endpoint model

The Bayesian linear model, outlined below will be used to model all continuous endpoints. This includes protocol estimands 17-34. In addition to the stratification factors, other prognostic covariates may be included in the model. The model estimates the posterior distribution of the linear effect of the IVIg arm compared to the SoC arm on the endpoint. We model the continuous endpoint, $Y_{ijkl} \in \{\mathbb{R}\}$, using a Gaussian distribution such that,

$$Y_{ijkl} \sim \mathrm{N}(\eta_{ijkl}, \sigma^2) \qquad \forall i \in \boldsymbol{I}, j \in \boldsymbol{J}, k \in \boldsymbol{K}, l \in \boldsymbol{L}$$

$$\eta_{ijkl} = \alpha_{jl} + \beta_{k^*} \quad \forall i \in \boldsymbol{I}, j \in \boldsymbol{J}, k^* \in \{\boldsymbol{K} | k > 1\}, l \in \boldsymbol{L}$$

Weakly informative prior distributions will be specified on the parameters α_{jl}, β_{k^*} and σ_l.

Binary endpoint model

The Bayesian logistic model, outlined below will be used to model all binary endpoints (estimands 2, 3-5, 9-10, 12-13, 14, 16 and 35-36). In addition to the stratification factors, other prognostic covariates may be included in the model. The model estimates the posterior distribution of the log odds ratio of the endpoint for the IVIg arm compared to the SoC arm. We model the binary endpoint, $Y_{ijkl} \in \{0,1\}$, using a Bernoulli model with a logistic link function such that,

$$Y_{ijkl} \sim \mathrm{Bernoulli}(\pi_{ijkl}) \quad \forall i \in \boldsymbol{I}, j \in \boldsymbol{J}, k \in \boldsymbol{K}, l \in \boldsymbol{L}$$

$$\pi_{ijkl} = \mathrm{logit}^{-1}(\eta_{ijkl}) \qquad \forall i \in \boldsymbol{I}, j \in \boldsymbol{J}, k \in \boldsymbol{K}, l \in \boldsymbol{L}$$

$$\eta_{ijkl} = \alpha_{jl} + \beta_{k^*}\ \forall i \in \boldsymbol{I}, j \in \boldsymbol{J}, k^* \in \{\boldsymbol{K} | k > 1\}, l \in \boldsymbol{L}$$

Weakly informative prior distributions will be specified on the parameters α_{jl} and β_{k^*}.

Time to event endpoint model

The Bayesian Weibull model outlined below will be used for the time to event endpoints (estimands 6, 7, 8, 11, 15, 37 and 39). In addition to the stratification factors, other prognostic covariates may be included in the model. The model estimates the posterior distribution of the log hazard of the endpoint for the IVIg arm compared to the SoC arm in each stratum $(j \times l)$. We model the time to event endpoint $\boldsymbol{t_{ijkl}} \in \{\mathbb{R}_{\geq 0}, \mathbb{Z}_2\}$ using:

$$t_{ijkl} \sim \text{Weibull}(\lambda_{ijkl}, \tau_{ijkl}) \quad \forall i \in \boldsymbol{I}, j \in \boldsymbol{J}, k \in \boldsymbol{K}, l \in \boldsymbol{L}$$

$$\lambda_{ijkl} = e^{\eta_{ijkl}} \quad \forall i \in \boldsymbol{I}, j \in \boldsymbol{J}, k \in \boldsymbol{K}, l \in \boldsymbol{L}$$

$$\eta_{ijkl} = \alpha_{jl} + \beta_{k^*} \quad \forall i \in \boldsymbol{I}, j \in \boldsymbol{J}, k^* \in \{\boldsymbol{K} | k > 1\}, l \in \boldsymbol{L}$$

with a hazard function $h(t_{ijkl}) = \lambda_{ijkl}\tau_{ijkl}t_{ijkl}^{\tau_{ijkl}-1} \quad \forall i \in \boldsymbol{I}, j \in \boldsymbol{J}, k \in \boldsymbol{K}, l \in \boldsymbol{L}$

Weakly informative prior distributions will be specified on the linear predictors α_{jl} and β_{k^*}, and on the shape parameter τ_{ijkl}. The appropriateness of the Weibull model will be evaluated using the property that, for a truly Weibull distributed survival time, the quantity $\log\left(-log\left(S(t_{ijkl})\right)\right)$ is approximately linear with respect to $\log(t_{ijkl})$, where $S(t_{ijkl})$ is the probability of bring alive at t_{ijkl}.

Exploratory model

The linear predictor η_{ijkl} from the primary model is extended to account for stratum specific effects:

$$\eta_{ijkl} = \alpha_{jl} + \beta_{jlk^*} + \sum_{p=1}^{P} \omega_{ip}\gamma_p \quad \forall i \in \boldsymbol{I}, j \in \boldsymbol{J}, k^* \in \{\boldsymbol{K} | k > 1\}, l \in \boldsymbol{L}$$

A hierarchical structure will be imposed on the stratum specific effects of the intervention β_{jlk^*} as it is anticipated that the ordinal outcome distributions may be mutually informative across graft type and age group strata. The parameter of interest β_{jlk^*} is then the common log-odds ratio that a participant in stratum $j \times l$ allocated to the IVIg arm has a higher ordinal outcome than a participant in the same stratum but allocated to the SoC arm.

There is a two-level hierarchical structure over the common log-odds ratios for participants allocated to the IVIg arm. For the **first level**, information is borrowed **across age groups** within graft type and for the **second level**, information is borrowed across **graft type**. The hierarchical structure is governed by the parameter vector $\{\mu_{jk^*}, \tau_{jk^*}, \mu_{k^*}, \tau_{k^*}\}$:

First level (borrowed across age groups): $\beta_{jlk^*} \sim \mathrm{N}\left(\mu_{jk^*}, \tau_{jk^*}^2\right) \ \forall j \in \boldsymbol{J}, k^* \in \{\boldsymbol{K}|k > 1\}, l \in \boldsymbol{L}$

Second level (borrowed across graft type): $\mu_{jk^*} \sim \mathrm{N}\left(\mu_{k^*}, \tau_{k^*}^2\right) \quad \forall j \in \boldsymbol{J}, k^* \in \{\boldsymbol{K}|k > 1\}$

The weakly informative prior distributions are:

$$\mu_{k^*} \sim \mathrm{N}(0, 1) \qquad \forall k^* \in \{\boldsymbol{K}|k > 1\}$$

$$\tau_{jk^*} \sim \mathrm{IG}(3,1) \quad \forall j \in \boldsymbol{J}, k^* \in \{\boldsymbol{K}|k > 1\}$$

$$\tau_{k^*} \sim \mathrm{IG}(3,1) \qquad \forall k^* \in \{\boldsymbol{K}|k > 1\}$$

The prior distributions on the standard deviation terms place the mode of the standard deviations around 0.25 with a weight of 2.3 (i.e., weakly informative with low density mass close to zero, thus information sharing across age groups or interventions will be data driven). The prior distributions for the stratum specific intercepts, covariate terms and cut points are the same as in the primary model.

Statistical Quantities

Quantities of interest

The quantities of interest are the common odds ratio of a higher ordinal outcome for the IVIg arm compared to the SoC arm measured 12 weeks after randomization. The quantities of interest will be derived from the primary estimand using the primary model. Model parameter (β_{k^*}) posterior probability densities will be employed to inform trial adaptation decisions and to report to the DSMB, in addition to quantifying intervention effects in any trial publications. Quantities of interest within each stratum will be reported to the DSMB in the unblinded reports.

Superiority

The trial will be stopped for superiority if the respective quantity of interest meets the decision rule below,

$$\mathrm{P}\left(e^{\beta_{k^*}} < \delta\right) > \epsilon_{\mathrm{supr}}$$

Here, $\delta = 0.9$ represents a minimum clinically significant difference and $\epsilon_{\mathrm{supr}} = 0.95$ is the superiority threshold. In other words, the trial will be stopped if the probability that the common odds ratio is less than 0.9, is greater than 0.95. If the decision rule is met, then IVIg will be declared superior to SoC.

Futility

The trial will be stopped for futility if the respective quantity of interest meets the decision rule below,

$$\mathrm{P}\left(e^{\beta_{k^*}} < \delta\right) < \epsilon_{\mathrm{futi}}$$

Here, $\delta = 0.9$ is the same minimum clinically significant difference as in the stopping rule for superiority and $\epsilon_{\mathrm{futi}} = 0.1$ is the futility threshold. In other words, the trial will be stopped if the probability that the common odds ratio is less than 0.9, is less than 0.1. If the decision rule is met, then the trial will be declared futile.

Sequential Analyses (Interims) and Trial Adaptations

Frequency and timing of sequential analyses (interims)

The first analysis will be performed after 100 eligible participants have completed 12 weeks of follow-up post randomization ("completers"); thereafter, analyses will be performed every 60 additional completers for the remainder of the trial until the maximum recruitment of 280 participants is reached.

Evaluation and reporting of decision criteria

The trial will involve regular sequential analyses, efficiently assessing the pre-planned decision criteria based on the accruing body of evidence, to answer research and policy questions and minimise the time until public disclosure of results. The pre-planned adaptations include stopping the trial when the decision rule (superiority or futility) is met for the primary estimand. After every analysis, the unblinded statistical team will prepare an unblinded report for the DSMB.

Simulations and Sample Size

The objective of the simulations is to understand the operating characteristics of the BEAT-BK trial and use these to justify: (i) maximum sample size; (ii) number and timing of interim analyses; and (iii) decision thresholds that maintain an appropriate level of type one error and power. This report is based on the status of the trial simulations on June 10, 2022.

The primary estimand, primary model and prior distributions for primary model parameters, and decision rule are described in the sections above. These trial simulations are generated for the two arms, standard of care alone (SoC) and standard of care plus IVIg (IVIg).

The following assumptions are made for the simulations:

- 5% loss to follow up 12 weeks after randomization.
- Full accrual, maximum of 280 participants, within the 4 years, assuming initial ramping up over the first year and uniform over years 2-4.
- Interims start when 100 participants have completed 12 weeks follow-up and are then performed each additional 60 participants.
- 5,000 trials were simulated for each scenario.
- The trial is stopped and IVIg is declared superior to SoC if $\mathbf{P}\left(e^{\beta} < \mathbf{0.9}\right) > \mathbf{0.95}$.
- The trial is stopped and declared futile if $\mathbf{P}\left(e^{\beta} < \mathbf{0.9}\right) < \mathbf{0.1}$.
- For simplicity and ease of interpretation, all simulations were generated without covariates.

The results of the simulations are presented in Table S2 and Figure S1. A maximum sample size of 280 participants, allowing for up to 5% loss to follow-up, is required to declare IVIg superior in over 88% of the simulations to detect a common odds ratio of 0.5 while maintaining type one error below 5% (Table S2). Additional gains in power are anticipated by the inclusion of covariates in the primary model. Figure S1 shows that the trial operating characteristics are robust to the plausible range of ordinal outcome distributions for the SoC arm. Given the limitations on feasible recruitment targets in this rare condition, clinical decision-making may still be guided for common odds ratios of 0.6 or lower (indicated by the green lines on Figure S1). The false positive rate (indicated by the blue lines on Figure S1) is controlled at approximately 5%.

Table S2. Proportion of simulated trials stopping for superiority or futility and mean sample sizes.

Simulation scenario	SoC Arm Ordinal Outcome Distribution						Proportion of trials stopping for superiority	Proportion of trials stopping for futility	Mean sample size
		1	2	3	4	5			
**1**: Null	A	20%	20%	20%	20%	20%	0.043	0.329	N = 241
(OR = 1)	B	25%	25%	25%	15%	10%	0.052	0.292	N = 245
	C	30%	30%	25%	12%	3%	0.065	0.254	N = 248
**2**: Small	A	20%	20%	20%	20%	20%	0.379	0.033	N = 240
(OR = 0.7)	B	25%	25%	25%	15%	10%	0.443	0.025	N = 233
	C	30%	30%	25%	12%	3%	0.461	0.015	N = 231
**3**: Moderate	A	20%	20%	20%	20%	20%	0.659	0.010	N = 212
(OR = 0.6)	B	25%	25%	25%	15%	10%	0.680	0.005	N = 207
	C	30%	30%	25%	12%	3%	0.698	0.006	N = 204
**4**: Large	A	20%	20%	20%	20%	20%	0.884	0.002	N = 176
(OR = 0.5)	B	25%	25%	25%	15%	10%	0.901	0.002	N = 171
	C	30%	30%	25%	12%	3%	0.900	<0.001	N = 170

Figure S1. Proportion of simulated trials declaring IVIg superior (as per the superiority condition) at each interim analysis by scenario and SoC arm ordinal outcome distribution. The red (black) dashed line represents 5% type one error (80% power).

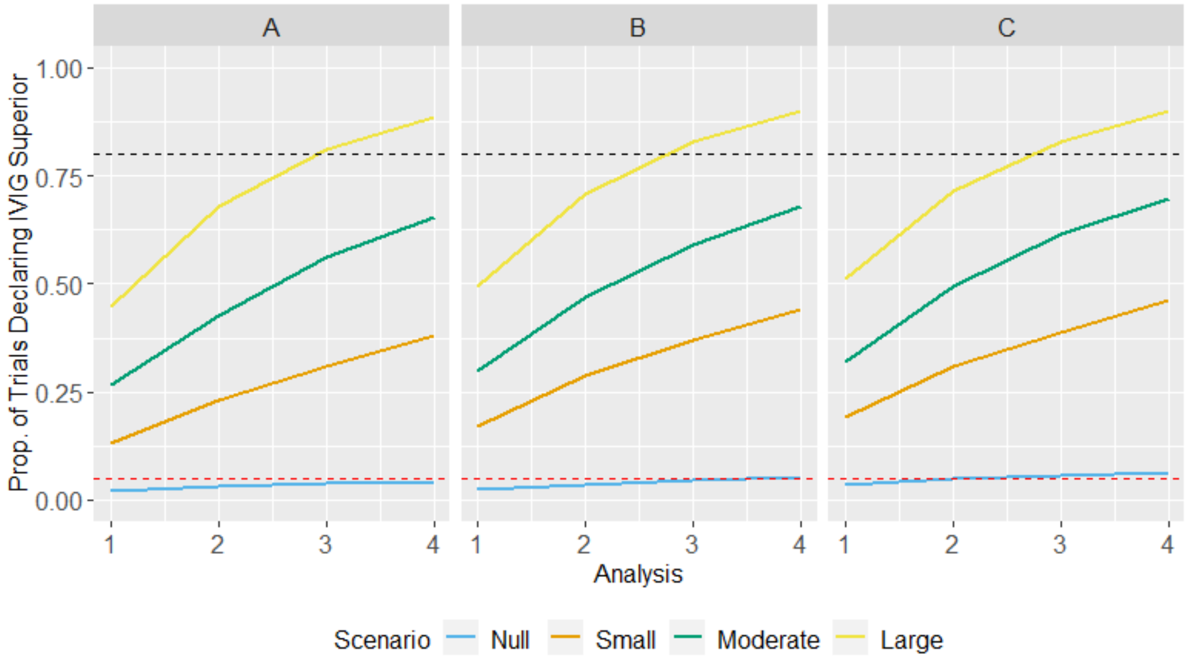

Predefined Adverse Events of Interest Associated with IVIg

Table S2. Predefined Adverse Events of Interest Associated with IVIg

Immediate Infusion Reactions (Events thought related to IVIg)
• Anaphylaxis or anaphylaxis-like reactions
• Transfusion related acute lung injury
• Pulmonary oedema
• Muscle pain or systemic influenza-like symptoms
• Headache/migraine
• Chills
• Fever
• Abdominal pain
• Flushing
• Nausea
• Chest tightness
• Mild wheezing
Delayed Infusion Reactions (events may or may not be related to the IVIg)
• Thromboembolic events including pulmonary emboli, deep vein thrombosis, superficial
thrombus at site of infusion
• Electrolyte disturbances including hyponatraemia and hypokalaemia
• Haemolysis
• Neutropenia
• Aseptic meningitis
• Acute kidney injury
• Skin rashes including urticaria, spot papule, eczema, pompholyx, lichenoid dermatitis, desquamation
Infections
• Bacterial
• Viral
• Fungal

• Protozoal
• Other
